# Anti-RBD immunoglobulin levels and their predictive value for SARS-CoV-2 neutralization

**DOI:** 10.1128/spectrum.02148-24

**Published:** 2025-04-08

**Authors:** Sabine Lichtenegger, Sissy Therese Sonnleitner, Sabine Saiger, Andrea Zauner, Melina Hardt, Barbara Kleinhappl, Gabriel E. Wagner, Ivo Steinmetz

**Affiliations:** 1 Diagnostic & Research Institute of Hygiene, Microbiology and Environmental Medicine, Diagnostic and Research Center for Molecular Biomedicine, Medical University of Graz 670030 , Graz, Austria; 2 Diagnostic & Research Institute of Pathology, Diagnostic and Research Center for Molecular Biomedicine, Medical University of Graz 31475, Graz, Austria; City of Hope Department of Pathology, Duarte, California, USA

**Keywords:** SARS-CoV-2, hybrid immunization, neutralization assay, anti-RBD binding antibodies, correlate of protection

## Abstract

A large number of studies have demonstrated that anti-receptor-binding domain (RBD)-binding antibody titers correlate with SARS-CoV-2 neutralization and protection from the disease. Unlike live virus neutralization assays, antibody-binding assays are easier to perform, require lower biosafety levels, and have therefore served as a substitute for virus neutralization assays throughout the SARS-CoV-2 pandemic. Although anti-RBD antibodies are usually neutralizing, there is evidence that they can also be non-neutralizing. Moreover, different immunization regimens can vary in the induction of SARS-CoV-2-neutralizing antibodies. In this study, we hypothesized that sera from individuals with different immunization and infection histories, but with the same amount of anti-RBD total immunoglobulin, differ in their neutralizing potency. A total of 27 sera from SARS-CoV-2 convalescent, vaccinated individuals, and 27 vaccinated-only individuals were investigated by using a widely used antibody-binding assay (Elecsys anti-SARS-CoV-2 S enzyme-linked immunosorbent assay [ELISA]) and a live virus neutralization assay. As expected, anti-RBD immunoglobulin units correlated with virus neutralization capacity within the vaccine and hybrid immunized group. However, sera from both groups with matched anti-RBD units varied significantly in their neutralization potential. In detail, our data indicate a significantly higher neutralization potency of hybrid immunity compared to vaccinated-only sera with similar anti-RBD immunoglobulin levels. Our study highlights the need for cautious interpretation of quantitative antibody data from anti-RBD ELISAs, especially when comparing differently immunized groups. In other words, very similar anti-RBD levels can show very different functional activity. This finding has implications for determining possible future correlates of protection.

**IMPORTANCE:**

Throughout the SARS-CoV-2 pandemic, neutralizing antibody levels have been central to predict a protective immune response. Anti-receptor-binding domain (RBD) enzyme-linked immunosorbent assays (ELISAs) correlate with neutralization assays and are due to the integration of simple performance with timely results used as surrogate assays. However, previous studies determining correlation used homogeneous cohorts. We reevaluated the correlation of a frequently used anti-RBD ELISA and a live virus neutralization assay using a heterogeneous cohort consisting of a vaccinated group without prior SARS-CoV-2 infection and a vaccinated convalescent group. The neutralizing capacity of sera with matched anti-RBD units significantly differed between groups, decreasing the correlation of the assays. Our findings highlight the necessity of considering the immunization context when interpreting serological tests and suggest that different immunization groups may require distinct protective thresholds. Considering the immunization history, we can develop more accurate predictions of immunity not only for SARS-CoV-2 but also for future challenges.

## INTRODUCTION

The SARS-CoV-2 pandemic was officially declared on 11 March 2020 (1). Quickly, countermeasures became available. As of December 2020, vaccines were administered on a large scale with initially high reported vaccine efficacy. However, the World Health Organization reports 774,954,393 COVID-19 cases claiming 7,040,264 deaths as of 4 April 2024 (2).

Assessing the immune status against SARS-CoV-2 is essential for introducing and adapting effective vaccination strategies and optimizing public health responses. Correlates of protection serve as efficient tools for these analyses with the measured parameters either directly influencing protection or correlating with it. Since specific antibodies are often directly involved in protection from disease, they are frequently used as these correlates for different viral diseases. Additionally, antibodies can be easily measured in serum samples. In line, SARS-CoV-2-neutralizing antibodies correlate with protection from COVID-19 (3, 4), and the introduction of an international standard facilitated interlaboratory comparability of assays for neutralizing antibody quantification (5–9).

The SARS-CoV-2 receptor-binding domain (RBD) of the viral spike protein directly mediates attachment to the host cell by binding to the eukaryotic angiotensin-converting enzyme-2 receptor. A significant proportion of neutralizing antibodies target this domain (10). In mechanistic terms, antibodies specific to the RBD domain block interactions with its cellular receptor and thus prevent subsequent virus entry. Therefore, it is hypothesized that assays quantifying anti-RBD-binding antibody levels align closely with neutralization assays, thus offering streamlined and expeditious diagnostic tools to predict the neutralizing potency of a serum. Unlike live virus neutralization assays, antibody-binding assays are easier to perform, require lower biosafety levels, and have served as hypothetical correlates of protection throughout the SARS-CoV-2 pandemic (5). Importantly, they can be carried out in high throughput settings. Most studies assessing the correlation of anti-RBD units and neutralizing antibodies focused on sera from a homologous group, for example, convalescent individuals or individuals vaccinated with a distinct vaccine (5–8). However, several studies suggest that vaccinated individuals show a higher proportion of non-neutralizing antibodies, and recently, it became evident that these non-neutralizing antibodies can also target the RBD region of the virus (11). Furthermore, neutralizing antibodies targeting the N-terminal domain and other regions of the spike protein exist, which are not detected by anti-RBD enzyme-linked immunosorbent assays (ELISAs).

We, therefore, hypothesized that sera with matched anti-RBD units differ in their neutralizing potency and investigated a heterogeneous cohort consisting of hybrid-immunized (vaccination post-COVID-19 recovery) and fully vaccinated individuals. Despite the detected correlation of the mentioned assays, our study shows that sera with similar anti-RBD-binding units can vary in their potency to neutralize SARS-CoV-2. This variation correlates with the immunization regimen, with the vaccinated-only cohort exhibiting a notably decreased neutralizing potency index (neutralization/antibody binding).

## MATERIALS AND METHODS

A total of 27 sera from SARS-CoV-2 convalescent, vaccinated individuals (13 with ChAdOx1-S [AstraZeneca], 13 with BNT162b2 [Pfizer], and 1 with mRNA-1273 [Moderna]) and 27 vaccinated-only individuals (CoVVac cohort [NCT04858607], two doses of mRNA-1273 [Moderna]) were investigated. For convalescent individuals, infection was confirmed by viral RT-PCR between March 2020 and December 2020, before the emergence of the SARS-CoV-2 Alpha (B.1.1.7) and Beta (B.1.351) variants. Characteristics of these cohorts were previously described (5, 12–14). Our study was conducted according to the guidelines of the Declaration of Helsinki and was approved by the Ethical Review Committee of the Medical University of Graz (EK 33–195 ex 20/21 and EK 1128/2021).

All used sera were heat-inactivated at 56° C for 30 min prior to use. Anti-RBD-binding units were determined by the Elecsys anti-SARS-CoV-2 S ELISA (ACOV2S, Roche Diagnostics, Mannheim, Germany) according to the manufacturer’s protocol and were previously published as summarized data in Sourij et al. (vaccinated-only group) (12) and Niedrist et al. (hybrid immunity group) (15).

Tissue culture infectious dose 50 (TCID_50_)-based neutralization assays were performed as previously described (5). Briefly, serum samples were twofold serially diluted (1:5–1:10,240) and mixed with the SARS-CoV-2 isolate BetaCoV/Munich/BavPat1/2020, which was obtained from a clinical case in Germany diagnosed after returning from China (European Virus Archive Global # 026 V-03883). An immunoglobulin-depleted serum served as a control. After 1 hour of incubation, quadruplicates of the same virus:serum (50  µL serum and 6  µL virus) mixture were transferred to 96-well plates containing 12,000 Vero E6 (ATCC CRL-1586) cells/well seeded the day before the assay in minimum essential medium (MEM, ThermoFisher Scientific, Austria) without fetal calf serum (FCS). Before seeding, Vero E6 cells were cultured in MEM supplemented with 10% FCS (ThermoFisher Scientific, Austria) at 37° C with 5% CO_2_. A multiplicity of infection of 0.0025 was used, as determined by immunoplaque assay. After 48 hours at 37° C, the cytopathic effect was evaluated under a light microscope. Neutralization titers (NT_50_) of sera reflect the highest serum dilution at which 50% of wells were protected from virus-induced cytopathic effects compared to a 100% control (immunoglobulin-depleted serum).

Virus propagation was performed as previously described (5). Vero E6 cells were infected, and the supernatant was harvested 72 hours after infection, clarified by 0.2  µm filtration, and frozen at −80°C before further use. Conventional immunoplaque assay was subsequently used for virus quantification (5). Briefly, Vero E6 cells were seeded in 48-well plates and after 24 hours infected with 200 µL virus. Wells were washed with 200 µL MEM medium after 1 hour at 37°C and overlaid with 1.5% carboxymethylcellulose in MEM. After three days at 37°C in 5% CO_2_, 4% paraformaldehyde in phosphate-buffered saline (PBS) was applied as fixative for 30 min at room temperature. Cells were washed with PBS and permeabilized with 0.1% Triton X-100 (Merck, Austria) in PBS for 10  min. After a subsequent washing step with PBS, cells were incubated in 3% H_2_O_2_ in methanol. Cells were washed with PBS and incubated with the primary antibody (anti-nucleoprotein, 40143-R019, SinoBiological) for 1 hour at room temperature. The secondary antibody (anti-rabbit, Agilent Technology, Dako, Austria) was added, and cells were incubated for 30  min. After incubation with chromogen for 1 min, plaques were analyzed by microscopy (Agilent Technology, Dako, Austria).

All work with virus isolates was performed in a Class II Biosafety Cabinet under BSL-3 conditions at the Diagnostic and Research Institute of Hygiene, Microbiology and Environmental Medicine, Medical University of Graz. Statistical analysis was performed using the Mann–Whitney U test. Significance was determined as *P* < 0.05. Analyses were conducted using default parameters in GraphPad Prism (version 9). Graphs were drawn with GraphPad Prism (version 9). A post hoc power analysis was performed using IBM SPSS Statistics 29.0.0.0 indicating that with 27 samples per group, our study had 60% power to detect a medium effect size (Cohen’s *d* = 0.5) at a significance level of 0.05.

## RESULTS

**Fig 1:**
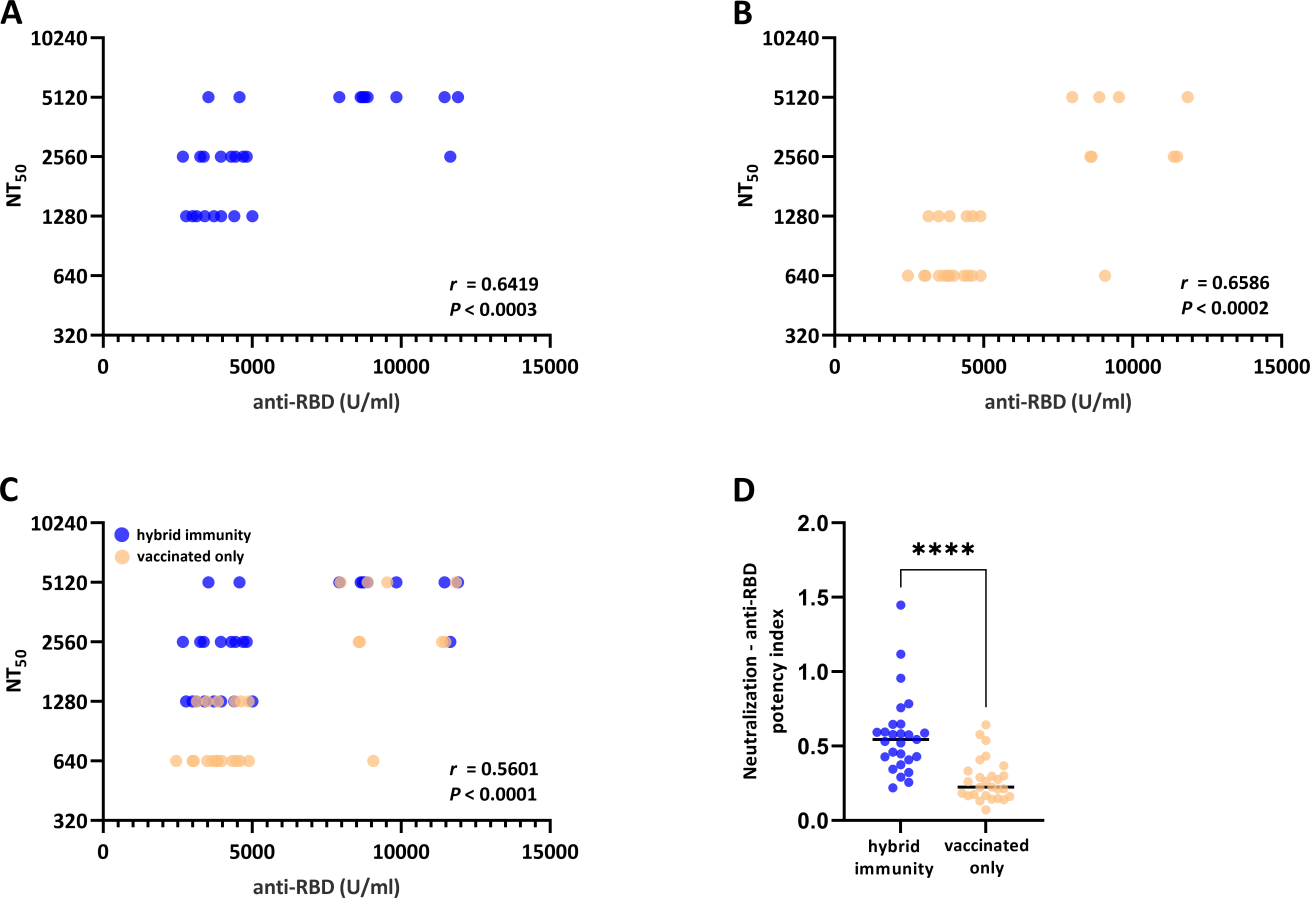
Anti-RBD-binding antibodies (total Ig) and viral neutralization capacity in vaccinated individuals with prior SARS-CoV-2 infection (hybrid immunity group) and without prior SARS-CoV-2 infection (vaccinated only). Chart of anti-RBD Ig units (abscissa) measured by Elecsys Anti-SARS-CoV-2S ELISA versus NT_50_ values (ordinate) measured by the TCID_50_-based neutralization assay for hybrid-immunized individuals (A), vaccinated-only individuals (B) as well as a mixed population (C). Virus neutralization:anti-RBD potency index calculated as the ratio of NT_50_ to anti-RBD units. Correlations in A–C show Spearman's rank correlation with two-tailed *P* values. Significant differences were determined by the Mann–Whitney U test. ****P < 0.0001 (D). U = units. *n* = 54.

The aim of this study was to assess whether sera from different immunization regimens with similar amounts of anti-RBD-binding antibodies show the same or a different proportion of neutralizing antibodies. Specifically, we used sera drawn from vaccinated-only and hybrid-immunized individuals. All sera were tested for anti-RBD total Ig-binding units using the Elecsys anti-SARS-CoV-2 S ELISA (Roche Diagnostics, Mannheim, Germany), a widely used standard assay. Hybrid-immunized sera were matched with vaccinated-only sera according to these units. SARS-CoV-2 neutralization was tested using a live virus neutralization assay with an original SARS-CoV-2 strain for the following reasons: the anti-RBD ELISA and the vaccines were designed with the initial SARS-CoV-2 strain, and convalescent sera were collected in the first wave of SARS-CoV-2 and before the emergence of variants. Fig. 1 shows that anti-RBD units correlate with virus neutralization for the hybrid immunity group (Fig. 1A, *r* = 0.6419) and the vaccination-only group (Fig. 1B, *r* = 0.6586). However, this correlation is weaker when both groups are combined (Fig. 1C, *r* = 0.5601). In fact, vaccination-only sera show significantly decreased neutralization potency (NT:anti-RBD units, Fig. 1D). Taken together, these data demonstrate that sera with matched anti-RBD units vary significantly in their neutralizing potency.

## DISCUSSION

Determining the immune status against infectious diseases, such as SARS-CoV-2, holds paramount importance for public health, facilitating the evaluation of vaccination coverage rates within populations. This insight enables the assessment of vaccination campaign efficacy, identification of demographics with suboptimal vaccination coverage, and targeted implementation of measures to enhance protection, particularly among vulnerable groups. Furthermore, monitoring the immune status facilitates outbreak surveillance, health resource planning, and evidence-based intervention development to mitigate disease spread at a population level.

While live virus neutralization tests are the gold standard for assessing the virus-neutralizing activity of a serum, their widespread application is impeded by logistical challenges. These tests necessitate BSL-3 facilities, are resource-intensive, and lack suitability for rapid mass screening due to their complexity. Hence, serological high-throughput methods, such as ELISAs and chemiluminescent immunoassays, are preferred in routine diagnostic settings. Consequently, we and others previously investigated whether antibody-binding assays correlate with virus neutralization as assessed by the gold standard, live virus neutralization assays. These studies demonstrated that there is a strong correlation between anti-RBD total Ig binding units measured by ELISA and neutralizing antibodies measured by live virus neutralization assays (5, 9, 16). This observation suggests that measuring anti-RBD-binding units could effectively serve as a proxy for quantifying neutralization capacity.

However, recent evidence suggests that non-neutralizing antibodies also target the RBD domain of the virus (11, 17). Therefore, we questioned whether anti-RBD-binding antibodies can indeed always serve as predictors for the neutralizing potency of a serum. This study shows that anti-RBD-binding units significantly correlate with SARS-CoV-2 neutralization for all groups tested. However, we detect variations in the NT_50_ of sera with matched anti-RBD units ranging, for example, between 640 and 5,120 for the group with anti-RBD units between 2,500 and 5,000 U/ml (Fig. 1C). This correlated with the immunization regimen, meaning that the neutralization potency (virus neutralization:anti-RBD units) of a serum is increased in the hybrid immunity group compared to the vaccination-only group. This discrepancy might be the result of a higher proportion of non-neutralizing antibodies in the vaccination-only group, which is in line with a study by Amanat et al. (17). Amanat et al. report that post-vaccination sera contain a high proportion of non-neutralizing antibodies, some of which bind to the RBD domain of the virus.

A notable limitation of our study is the representation of different vaccination regimens in the hybrid-immunized and vaccinated-only groups. Therefore, we cannot entirely exclude that the significantly higher potency index for the hybrid immunized group is based on different immunization regimes, which should be further evaluated in future studies. However, this does not affect the key observation of our study, specifically that sera from different cohorts with matched anti-RBD levels can show very different functional activity. While we further acknowledge the limitations of our sample size, we emphasize the importance of our findings. In the context of potential future SARS-CoV-2 outbreaks, it is crucial to recognize that RBD titer levels do not necessarily reflect the quality of the antibody neutralization capacity of a serum. Therefore, they should not be directly considered as reliable correlates of protection without taking the immunization history into account.

Our findings indicate that although anti-RBD immunoglobulin levels correlate with neutralizing antibody levels, sera with similar anti-RBD units can exhibit varying neutralization potencies depending on the immunization regime. This underscores the importance of considering immunization history in serological assessments. Given the ongoing evolution of SARS-CoV-2, the issure remains of high relevance. Discrepancies between anti-RBD binding and neutralizing potency may persist or worsen with variants carrying RBD mutations, including the currently circulating ones. Such mutations can alter antibody binding and neutralization, complicating the use of anti-RBD-binding assays to predict neutralization. This underscores the need to cautiously interpret anti-RBD ELISA results and adapt and reevaluate serological assays to the evolving antigenic landscape of SARS-CoV-2.

### Conclusions

The SARS-CoV-2 pandemic has emphasized the critical requirement for serological tests that are amenable to routine use during acute outbreak scenarios and accurately represent individual protective levels. Since anti-RBD antibodies usually neutralize the virus and strongly correlate with neutralization, they are discussed as correlates of protection. However, our concise investigation reinforces that assays targeting RBD-specific antibodies do not necessarily predict a specific neutralization titer. Our findings underscore the importance of considering the immunization context when interpreting serological assays across different cohorts and predicting protective immunity.
